# Alveolar rhabdomyosarcoma has superior response rates to vinorelbine compared to embryonal rhabdomyosarcoma in patients with relapsed/refractory disease: A meta‐analysis

**DOI:** 10.1002/cam4.5749

**Published:** 2023-04-04

**Authors:** Wendy Allen‐Rhoades, Philip J. Lupo, Michael E. Scheurer, Yueh‐Yun Chi, John F. Kuttesch, Rajkumar Venkatramani, William H. Meyer, Leo Mascarenhas

**Affiliations:** ^1^ Department of Pediatric and Adolescent Medicine Mayo Clinic Minnesota Rochester USA; ^2^ Department of Pediatrics Baylor College of Medicine Texas Houston USA; ^3^ Cancer and Blood Disease Institute, Children's Hospital of Los Angeles, Norris Comprehensive Cancer Center and Department of Pediatrics, Keck School of Medicine University of Southern California Los Angeles California USA; ^4^ Department of Pediatrics University of New Mexico Albuquerque New Mexico USA; ^5^ Department of Pediatrics University of Oklahoma Health Sciences Center Oklahoma City Oklahoma USA

**Keywords:** chemotherapy, clinical cancer research, clinical observations, pediatric cancer, soft tissue sarcoma

## Abstract

**Background:**

Patients with alveolar rhabdomyosarcoma (ARMS) have inferior outcomes compared to patients with embryonal rhabdomyosarcoma (ERMS) and more effective chemotherapy options are needed for these patients. Vinorelbine is a semisynthetic vinca alkaloid that has clinical activity in relapsed rhabdomyosarcoma (RMS) when used alone or in combination with cyclophosphamide.

**Aims:**

The goal of our study was to evaluate whether RMS histology subtype influences response rate to vinorelbine alone or in combination.

**Materials & Methods:**

Five Phase 2 trials that enrolled RMS patients were included in the meta‐analysis. Two studies evaluated vinorelbine alone, two studies evaluated vinorelbine in combination with low dose oral cyclophosphamide, and one study evaluated vinorelbine and intravenous cyclophosphamide in combination with temsirolimus or bevacizumab. All RMS patients had relapsed or refractory disease and had received at least one prior therapy. Response was reported according to RECIST1.1 and was defined as a complete or partial response. Response data was obtained from published results or from trial principal investigator. RMS NOS patients were grouped with ERMS patients for this analysis. Summary estimates comparing differences between ARMS and ERMS response rates were generated using a random‐effects model to account for heterogeneity among the studies.

**Results:**

One hundred fifty‐six enrolled patients evaluable for response were included in the meta‐analysis, 85 ARMS, 64 ERMS and 7 RMS‐NOS. The combined effect generated from the random‐effects model demonstrated a 41% increase (*p* = 0.001, 95% CI; 0.21–0.60) in response to vinorelbine as a single agent or in combination in patients with ARMS compared to patients with ERMS. There was no significant difference in the rate of progressive disease between patients with ARMS compared to ERMS (*p* = 0.1, 95%CI; −0.26–0.02).

**Discussion:**

Vinorelbine is an active agent for the treatment of relapsed or refractory RMS and a meta‐analysis of Phase 2 studies shows that radiographic responses in patients with ARMS were significantly higher than ERMS or RMS‐NOS.

**Conclusion:**

These data support further investigation of vinorelbine in newly diagnosed patients with RMS particularly those with alveolar histology.

## Graphical abstract

Meta‐analysis of 5 studies evaluating vinorelbine in patients with relapsed or refractory rhabdomyosarcoma demonstrated a 41% increase in response to vinorelbine for patients diagnosed with alveolar rhabdomyosarcoma compared to patients diagnosed with embryonal rhabdomyosarcoma or rhabdomyosarcoma NOS.

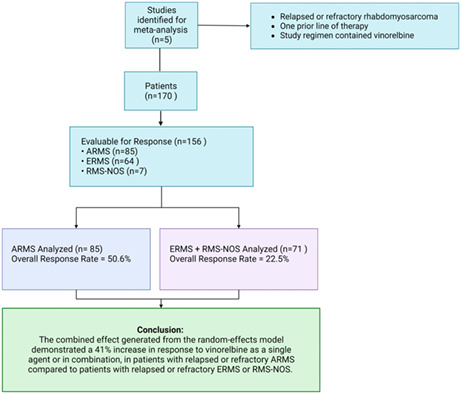

## 1 INTRODUCTION

Rhabdomyosarcoma (RMS) is the most common soft tissue sarcoma diagnosed in children and adolescents, with approximately 350 new patients diagnosed every year in the United States. Survival outcomes for RMS vary from 10% to 90% depending on histology, molecular translocation, stage, and clinical grouping.^1^, ^2^ Two main RMS histologic subtypes have been recognized for over 60 years and are conventionally termed embryonal RMS (ERMS) and alveolar RMS (ARMS). RMS can further be classified molecularly by the presence or absence of a chromosomal translocation into fusion negative RMS (FN‐RMS) or fusion positive (FP‐RMS). The most common translocations involve *FOXO1* with either *PAX3* or *PAX7* and are exclusively seen in the ARMS subtype.^3^

Patients with ARMS or FP‐RMS are more likely to present with metastatic disease and have worse prognosis, both at diagnoses and following relapse when compared to patients with ERMS or FN‐RMS.^4^ Improvements in overall survival have been achieved for patients with low and intermediate risk RMS, but patients with high‐risk disease continue to have poor survival outcomes. Standard treatment for RMS includes chemotherapy and local control of the primary tumor with surgery, radiation, or both. The standard chemotherapy backbone for the last 30 years for most RMS in North America has included vincristine, dactinomycin and cyclophosphamide (VAC) with variations in dose and intensity for the different clinical risk groups.

Several novel agents have been tested in Phase 1 and Phase 2 clinical trials in treatment naïve patients and patients with relapsed or refractory RMS with variable response rates, ranging from 0–70%.^5^, ^6^, ^7^, ^8^ However, to date, none have provided superior outcomes over VAC when tested in newly diagnosed RMS patients including those with high risk disease where new treatments are desperately needed. Vinorelbine (VINO) is a second generation semi‐synthetic vinca alkaloid that has a different site of structural modification than vincristine (one of the most active drugs in RMS), which leads to less neurotoxicity than vincristine.^9^, ^10^, ^11^, ^12^ VINO has been tested as a single agent and also in combination with cyclophosphamide in heavily pre‐treated patients with RMS with promising results.^13^, ^14^, ^15^, ^16^, ^17^ Most recently, the European Pediatric Soft Tissue Sarcoma Study Group (EpSSG) evaluated VINO and oral cyclophosphamide as maintenance chemotherapy in a large cohort of non‐metastatic RMS in the EpSSG RMS 2005 trial (NCT00339118).^18^ In that study, newly diagnosed patients with localized ERMS who achieved a complete remission (CR) benefitted from the addition of maintenance chemotherapy. It is unclear whether the improvement in overall survival noted on RMS 2005 was due to the addition of VINO to the regimen, the longer duration of therapy, or the delivery of metronomic oral cyclophosphamide.

To further evaluate the potential benefit of VINO in the treatment of patients with high‐risk RMS, we conducted a meta‐analysis of 5 early phase trials that utilized VINO and enrolled patients with relapsed and/or refractory RMS, and analyzed response rates to VINO in patients with ERMS and ARMS.

## 2 MATERIALS AND METHODS

### 2.1 Study selection

Studies were identified through literature search conducted in the NCBI PubMed/MEDLINE database using text word search strategies. The following search string was utilized (vinorelbine) AND (rhabdomyosarcoma). All prospective clinical trials that utilized VINO and had RMS patients with relapsed or refractory disease enrolled were included. Bibliographies of published articles were manually searched to identify additional studies. Previously unpublished histology specific response data from a phase 2 clinical trial (NCT01222715) that included the use of VINO in first relapse RMS was obtained from the COG clinical trials database.^16^

### 2.2 Data extraction

First, response data were obtained from published results and missing information obtained from trial principal investigator. Response was reported according to RECIST1.1.^19^ Complete response (CR) was defined as the disappearance of all target lesions, partial response (PR) was defined as at least a 30% decrease in the sum of diameters of target lesions, taking as reference the baseline sum of diameters with no evidence of progression in any lesion and no new lesions. Objective response rate (ORR) was defined as the percentage of CR and PR. Second, data on RMS histology was obtained from each study. Specifically, patients were classified according to histologic subtype as ARMS or ERMS. Patients with RMS‐not otherwise specified (RMS‐NOS) were grouped with ERMS patients for this analysis. Molecular translocation was not utilized for classification due to unavailability of information.

### 2.3 Statistical methods

First, we first tested for heterogeneity across studies using Cochran's Q‐test. As there was evidence of heterogeneity (*p* < 0.1), we used a random‐effects model applying the restricted maximum likelihood (REML) method, which provided a more robust summary effect estimate across heterogeneous study‐specific estimates. The random‐effects model was used to generate summary risk difference estimates (i.e., θ = ORR) by RMS histology (i.e., ARMS vs. ERMS). Specifically, as noted above we sought to determine if there were significant differences in ORR by RMS histology. Statistically significant summary ORRs were those where *p* < 0.05. Analyses were conducting using the meta commands in Stata v16 (College Station, TX).

## 3 RESULTS

Five Phase 2 trials that enrolled RMS patients were included in the meta‐analysis.^13^, ^14^, ^15^, ^16^, ^17^ 170 patients with relapsed or refractory RMS were enrolled and treated on the 5 studies. All patients had relapsed or refractory RMS and had received at least one prior line of systemic therapy. Two studies evaluated VINO alone, two studies evaluated VINO in combination with low‐dose oral cyclophosphamide, and one study evaluated VINO and intravenous cyclophosphamide in combination with temsirolimus or bevacizumab.^13^, ^14^, ^15^, ^16^, ^17^ VINO doses ranged from 15 mg/m^2^/dose to 33.75 mg/m^2^/dose and the most common dose was 25 mg/m^2^/dose. See Table 1 for trial specifics.

**TABLE 1:** Summary of trials included in meta‐analysis.

Trial	Enrollment	VINO Dose	VINO schedule	Other chemotherapy	Enrolled, evaluable
Casanova^15^	1998–2001	30 mg/m^2^/dose	Days 1 and 8 every 21 days	N/A	13, 12
Casanova^14^	2002–2003	15–30 mg/m^2^/dose	Days 1, 8 and, 15 every 21 days	Oral cyclophosphamide 25 mg/m^2^/day	9, 8
Kuttesch^13^	1998–2002	30–33.75 mg/m^2^/dose	Day 1 × 6 weeks, 2 week break	N/A	11, 10
Minard‐Colin^17^	2003–2008	25–30 mg/m^2^/dose	Days 1, 8, and 15	Oral cyclophosphamide 25 mg/m^2^/day	50, 48
Mascarenhas^16^	2010–2013	25 mg/m^2^/dose	Days 1 and 8	Cyclophosphamide 1200 mg/m^2^/dose on Day 1. PLUS one of the following: bevacizumab 15 mg/kg/dose on days 1, 8 and 15 OR temsirolimus 15 mg/m^2^/dose on days 1, 8 and 15	87, 78

A total of 170 eligible patients were enrolled, and 156 were evaluable for response. Of the 156 included in the analysis, 85 were diagnosed with ARMS, 64 were diagnosed with ERMS, and 7 were diagnosed with RMS‐NOS. Responses were assessed at every 6 weeks,^14^, ^15^ every 8 weeks,^13^, ^17^ and at weeks 6, 12, 18, 27 and 36.^16^ The ORR for VINO containing therapy for all patients was 37.8% (95% CI: 30.0–45.6%). Patients with ERMS/RMS‐NOS had an ORR of 22.5% (95% CI: 11.1–33.9%) and patients with ARMS had an ORR of 50.6% (95% CI: 40.2–61.0%) (Table 2).

**TABLE 2:** Histologic specific response rates.

Study	Casanova^15^	Casanova^14^	Kuttesch^13^	Minard‐Colin^17^	Mascarenhas^b^, ^16^		Total
Population	Relapsed Refractory	Relapsed Refractory	Relapsed Refractory	Relapsed Refractory	First relapse		
Therapy	VINO	VINO/Cyclo	VINO	VINO/Cyclo	VINO/Cyclo Bev	VINO/Cyclo Tem	
RMS Enrolled	13	9	11	50	44	42	169
ARMS^a^	6	1	5	25	24	24	85
CR/PR	5	1	3	12	9	13	43
ORR	—	—	—	—	—	—	50.6%
ERMS^a^	6	7	5	23	16	14	71
CR/PR	1	2	0	5	3	5	16
ORR							22.5%

Abbreviations: Bev, Bevacizumab; CR, complete response; Cyclo, Cyclophosphamide; ORR, Objective Response Rate; PR, partial response; Tem, Temsirolimus; VINO, Vinorelbine.

^a^ Assessable.

^b^ Histology specific response data is unpublished.

**FIGURE 1:**
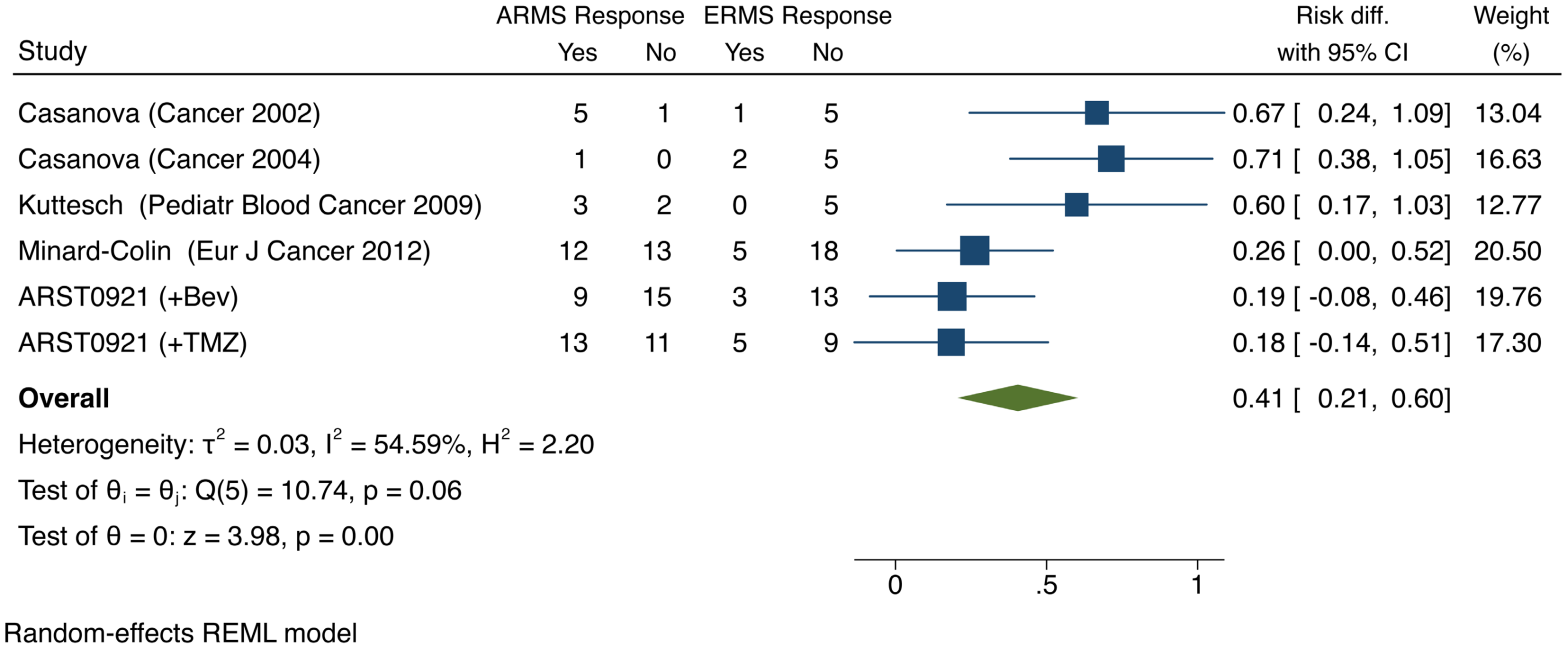
The combined effect generated from the random‐effects model shows a 41% increase in response to vinorelbine or vinorelbine containing regimens for patients with alveolar histology compared to patients with embryonal histology.

**FIGURE 2:**
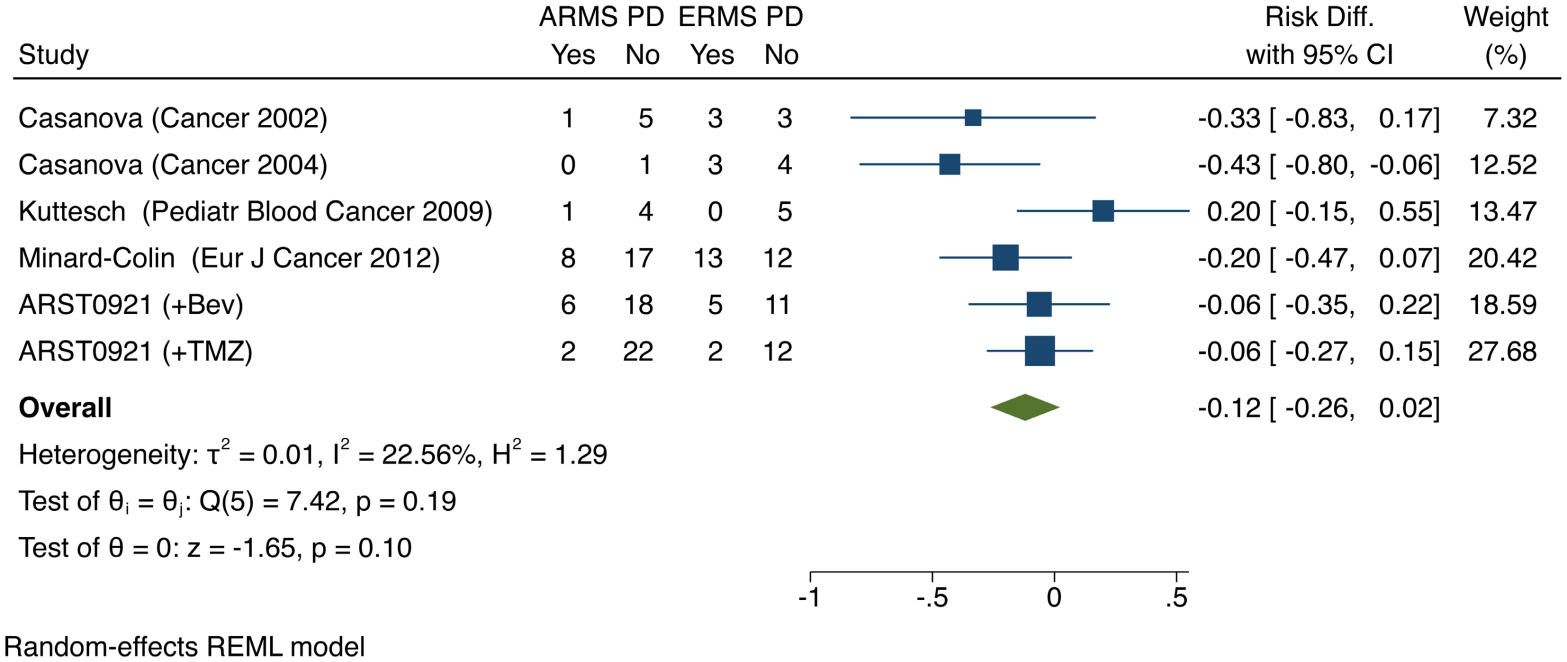
There was no significant difference in progressive disease for patients with ARMS compared to ERMS when treated with vinorelbine or vinorelbine containing regimens in the meta‐analysis.

The combined effect generated from the random‐effects model demonstrated a 41% increase (*p* = 0.001, 95% CI; 21–60%) in response to VINO as a single agent or in combination, in patients with ARMS compared to patients with ERMS or RMS‐NOS (Figure 1). There was no significant difference in the rate of progressive disease between patients with ARMS compared to ERMS (*p* = 0.1, 95% CI; −0.26‐2%) (Figure 2).

## 4 DISCUSSION

This meta‐analysis demonstrates a significantly higher objective response rate for patients with relapsed refractory ARMS when treated with VINO alone or in combination across five early phase clinical trials compared to patients with ERMS or RMS‐NOS. The United States Food and Drug Administration approved VINO for chemotherapeutic use in 1994.^10^ Since then, VINO has been extensively studied in children through Phase 1 and 2 trials.^13^, ^20^ The most commonly used dose and schedule is 25 or 30 mg/m^2^/dose administered intravenously weekly.^13^, ^20^ In addition, the safety and tolerability of VINO in combination with other chemotherapeutic agents in children has also been demonstrated.^14^, ^16^, ^21^, ^22^

An efficacy signal of VINO in RMS was noted 20 years ago.^17^ Two phase 2 clinical trials in heavily pre‐treated patients with RMS demonstrated single agent activity of VINO when given weekly at 30 mg/m^2^/dose. The ORR observed in these studies was 36% and 50%, including 1 CR and 9 PRs.^13^, ^15^ Similarly, a lower dose of VINO (25 mg/m^2^/dose) was evaluated in combination with daily oral cyclophosphamide in a larger cohort of heavily pre‐treated patients with RMS. Four CRs and 14 PRs were observed, with an ORR of 36%. These response rates are superior to those seen in other phase 2 trials for patients with relapsed RMS, and comparable to response rates with other agents tested in upfront phase 2 windows in treatment naïve patients.^5^, ^6^, ^23^ In patients with a first relapse of RMS, VINO combined with cyclophosphamide and bevacizumab or temsirolimus, produced a comparable ORR of 37%.^16^ However it should be noted that the current analysis of RMS response to VINO was not uniformly assessed at the same time point, nor did the publications report best response rates to VINO alone or in combination. Nevertheless, the significant difference in responses by histology remains noteworthy.

The EpSSG RMS 2005 trial investigated 6 months of VINO in combination with oral cyclophosphamide in as a maintenance regimen in newly diagnosed RMS patients with localized EpSSG‐defined high‐risk disease that attained a CR after 9 cycles of chemotherapy and local control (NCT00339118). This was the first trial in RMS to randomize patients to receive VINO with oral cyclophosphamide or not. The 3‐year disease free survival (DFS) and OS in the maintenance arm versus no maintenance were DFS 77.6% vs 69.8% (*p* = 0.0613) and OS 86.5% vs 73.7% (*p* = 0.0111), respectively.^18^ These results suggest that either the addition of VINO or the addition of maintenance chemotherapy improved OS for non‐metastatic RMS in CR at the end of nine cycles of chemotherapy.

The results of the current meta‐analysis show that in patients with heavily pre‐treated RMS, the response to VINO alone or in combination was 37.8%. The ORR observed in patients with relapsed or refractory ARMS was 50.6%, which is similar to the prior best response rate (48%) observed with vincristine and irinotecan in a phase 2 window in a patients with first relapse or refractory disease.^23^ Differential response rates by histology were also seen on that trial, with more patients with ARMS responding to vincristine and irinotecan compared to patients with ERMS or RMS‐NOS when utilizing the protracted irinotecan schedule, but not the shorter schedule. Further, patients with ERMS had a higher rate of non‐responsive disease when treated with the shorter irinotecan schedule (20% vs. 5%). There were no differences in survival outcomes of first relapse/refractory RMS patients treated with either the protracted or short schedule of irinotecan when administered with multi‐agent chemotherapy.^23^ However, histology specific survival outcomes were not reported on that study. In the current analysis, there were no observed differences in the rate of progressive disease between the patients with ARMS vs ERMS or RMS‐NOS. Together, these findings continue to underscore the differences in biology between ARMS and ERMS and potential importance of dose intensity and schedule.^23^

Studying VINO in patients with newly diagnosed high‐risk RMS is appealing for several reasons. First, VINO differs from vincristine in its structure, microtubule selectivity, and dose limiting toxicities. The chemical modification used to produce VINO allows it to bind more specifically to mitotic microtubules versus neural axonal microtubules, which leads to less neurotoxicity when compared to vincristine. Phase 1 studies confirm that the dose limiting toxicity of VINO is myelosuppression compared to neurotoxicity with vincristine.^9^, ^10^, ^12^ These differences in toxicity profile are particularly important for patients diagnosed with high risk disease as they tend to be older.^24^, ^25^ Compared to younger patients, older patients have increased risk of neurotoxicity from vinca alkaloids, which can lead to dose interruptions or reductions and may negatively affect outcomes.^26^ Second, the patients included in the current study were heavily pre‐treated with vincristine, indicating that VINO and vincristine are sufficiently different clinically that patients still garner benefit from re‐treatment of the same class of drugs. Lastly, the improved response rate to VINO observed in patients with ARMS compared to ERMS is important, as nearly 70% of patients with metastatic or high‐risk disease will have ARMS.^5^, ^25^

The addition of VINO to a regimen that includes vincristine may allow for vinca alkaloid intensification by combining first and second generation vinca alkaloids that mitigate toxicity overlap, which is mainly neurotoxicity. Theoretically, vinca alkaloid intensification could be clinically beneficial in two ways. First, continuous exposure to vinca alkaloids may be more efficacious because this class of drugs is mostly dependent on the length of exposure of the microtubules to the drug rather than high peak concentrations.^27^ Second, intensification will also minimize “time off” drug which could potentially reduce emergence of other drug resistance mechanisms such as the overexpression of drug efflux pumps such as P‐glycoprotein.^28^

Several limitations exist within this meta‐analysis, including specific information on number and type of relapse therapies received and patterns of relapse. In addition, the underlying biologic process that could explain the observed increased response rate to VINO for patients with ARMS compared to ERMS seen in this study is currently unclear. However, there are distinct molecular drivers between the two subtypes, with ARMS being driven by *FOXO1* fusion oncogenes and ERMS being largely driven by mutations in receptor tyrosine kinases and Ras superfamily (HRAS, NRAS, KRAS) and downstream targets.^29^ The current study was unable to assess the role of fusion status and relied solely on morphology to identify ARMS or ERMS. Furthermore, the historical rate of fusion negative ARMS is approximately 20% and the inability to evaluate response based on fusion status instead of histology is a limitation and should be evaluated prospectively to determine if fusion type influences response to VINO.^1^, ^30^, ^31^

VINO is now being investigated in an ongoing COG trial for patients with newly diagnosed high‐risk RMS (NCT04994132), as well as in another cooperative group trial conducted by the National Pediatric Cancer Foundation (NCT04388839). VINO has not been previously studied alone or in combination for patients newly diagnosed with HR‐RMS and treated on North American regimens. The COG intermediate risk trial ARST1431 (NCT02567435) included the addition of maintenance chemotherapy with VINO plus oral cyclophosphamide (identical to the EpSSG maintenance regimen) to both randomized arms. The trial recently closed to enrollment and results are currently unavailable. It will be challenging to assess the benefit of this addition of VINO given the trial design. However, the data presented within this report provides evidence that the addition of VINO to patients with newly diagnosed high‐risk RMS may benefit from the incorporation of VINO upfront and/or adding it during maintenance therapy as was done in the EpSSG RMS 2005 trial (NCT00339118).

## 5 CONCLUSIONS

Our analysis shows that VINO is an active agent to treat patients with relapsed or refractory RMS and patients with ARMS had significantly higher response rates compared to patients with ERMS or RMS‐NOS. This data, in part, provided rationale for including VINO in the cooperative group trial ARST2031 (NCT04994132), which was activated in September 2021 and is anticipated to enroll newly diagnosed patients with high‐risk RMS for approximately 30 months.

## AUTHOR CONTRIBUTIONS

**Wendy A Allen‐Rhoades:** Conceptualization (equal); data curation (equal); formal analysis (equal); project administration (lead); writing – original draft (lead); writing – review and editing (lead). **Philip J. Lupo:** Data curation (equal); formal analysis (equal); methodology (equal); validation (equal); writing – review and editing (equal). **Michael E. Scheurer:** Data curation (equal); formal analysis (equal); methodology (equal); validation (equal); writing – review and editing (equal). **Yueh‐Yun Chi:** Data curation (equal); formal analysis (equal); methodology (equal); validation (equal); writing – review and editing (equal). **John Kuttesch:** Writing – review and editing (equal). **Rajkumar Venkatramani:** Writing – review and editing (equal). **William H Meyer:** Writing – review and editing (equal). **Leo Mascarenhas:** Conceptualization (equal); data curation (equal); methodology (equal); supervision (lead); writing – original draft (supporting); writing – review and editing (equal).

## FUNDING INFORMATION

Research reported in this publication was supported by the St. Baldrick's Foundation, the Children's Oncology Group, The National Cancer Institute of the National Institutes of Health under award numbers U10CA180886 and U10CA180899. The content is solely the responsibility of the authors and does not necessarily represent the official view of the National Institutes of Health (NCI).

## CONFLICT OF INTEREST STATEMENT

WAR, RV, PJL, MES, WHM, JK, YYC have no conflicts of interest to declare. LM consulting (Bayer, Amgen) research funding to institution (Incyte, Jazz, Bayer, Pfizer, Eli Lily, ER Squib, Salarius, Turning Point, AstraZeneca, Bioatla).

## Data Availability

The response data supporting this meta‐analysis are from previously reported studies and datasets, which have been cited. The processed data are available from the corresponding author upon request.
